# An Observational Study on the Association Between the Curve of Spee and Sagittal Condylar Guidance in Different Skeletal Malocclusions

**DOI:** 10.7759/cureus.84679

**Published:** 2025-05-23

**Authors:** Mani M Tejashre, Shibitha Balakrishnan, Sai Snigdha Mandava, Gowri Sankar Singaraju, Jagath Kaushal Singaraju, Allu Harini, Prasad Mandava

**Affiliations:** 1 Orthodontics and Dentofacial Orthopedics, Narayana Dental College, Nellore, IND; 2 Orthodontics and Dentofacial Orthopedics, Malabar Dental College and Research Centre, Eddapal, IND; 3 Microbiology, Narayana Medical College, Nellore, IND

**Keywords:** condylar guidance, curve of spee, malocclusion, occlusal plane, orthodontic

## Abstract

Introduction

The Curve of Spee (COS) is integral to occlusal function and esthetics. Its depth and relationship with sagittal condylar guidance (CG) across various malocclusion classes remain underexplored, especially within the Indian demographic.

Objective

To evaluate and correlate the depth of the COS with CG and other craniofacial parameters in Class I, II, and III malocclusions.

Methods

An observational analytical study was conducted on 74 patients aged 18-25 years undergoing orthodontic treatment. Subjects were categorized into Class I (n=50), Class II (n=16), and Class III (n=8) based on the ANB angle. Assessed parameters included COS, CG angle (via orthopantomogram), cephalometric variables (SNA, SNB, ANB, FMA, FH-OP), and dental measures (overjet, overbite). Statistical analyses encompassed analysis of variance (ANOVA), Pearson’s correlation, and post hoc Tukey tests.

Results

COS differed significantly among malocclusion groups (p<0.001), being highest in Class II (2.98±0.95 mm) and lowest in Class III (1.75±0.4 mm). No significant difference was observed in CG across groups (p=0.464). A weak negative correlation existed between COS and CG in Class I and III, and a weak positive correlation in Class II, though not statistically significant.

Conclusion

COS significantly varies with sagittal malocclusion type. While condylar guidance does not significantly differ among groups, its weak correlation with COS suggests a potential but inconclusive relationship. Further research with larger samples and the inclusion of vertical growth patterns is warranted.

## Introduction

The curve of Spee (COS), a naturally occurring anteroposterior curvature of the human dentition, was first described by von Spee in 1890 and plays a key role in efficient mastication [1]. COS is typically measured as the vertical distance from the deepest cusp tip on the mandibular arch to the line connecting the mesiobuccal cusp of the mandibular second molar and the incisal edge of the most protruded lower incisor. Andrews described six keys to normal occlusion, of which the sixth pertains to the occlusal plane and emphasizes the importance of a flat or mildly curved COS for optimal intercuspation [2]. The occlusal plane is the subject of the sixth key to proper occlusion. To establish ideal static occlusion, it becomes necessary to level or slightly reverse the COS during orthodontic treatment to counteract its relapse tendency. Despite orthodontic correction, the COS may recur, indicating the need for controlled occlusal flattening during treatment planning. An ideal functional occlusion depends on maximum intercuspation coinciding with a stable centric relation, in which the condyles are seated in the glenoid fossa in the most superior and centered position [3]. Mandibular movement is governed by two terminal determinants: condylar guidance (CG), defined by the condylar path along the articular eminence, and incisal guidance (IG), which is dictated by the degree of overjet and overbite [4,5].

Numerous studies have explored the determinants of the COS, revealing consistent associations with dentofacial morphological parameters. Batham et al. and Patil et al. reported strong positive correlations between COS depth and sagittal discrepancies, especially overbite, overjet, ANB angle, and mandibular plane angle, with differences noted across malocclusion types [6,7]. Cheon et al. highlighted that overbite alone could account for over 25% of the variance in COS depth, with mandibular positioning and overjet also playing significant roles [8]. Several studies consistently highlight that Class II malocclusions, particularly Divisions 1 and 2, exhibit the deepest COS, while Class III malocclusions show the flattest curves [9-11]. The depth of COS has been positively associated with parameters such as overjet, overbite, ANB angle, and vertical skeletal pattern [12,13]. Rozzi et al. showed that the vertical growth patterns affect COS correction: low-angle patients responded with incisor intrusion, while high-angle cases showed posterior extrusion [14]. Effective reduction of COS in Class II Div 1 malocclusion non-extraction cases treated by reverse COS nickel-titanium (NiTi) wires occurred via lower premolar and molar extrusion [15]. Another study by Fawaz et al. linked deeper COS to hypodivergent skeletal patterns and molar angulation [16].

Despite extensive work on COS and its skeletal associations, limited evidence exists linking COS to CG. Understanding this relationship could improve orthodontic diagnosis, mechanics, and long-term stability in different skeletal malocclusions. Investigating this relationship could help anticipate occlusal changes during orthodontic correction in various skeletal malocclusions.

This study was taken up to evaluate and correlate the depth of the COS with sagittal CG and selected cephalometric parameters across Class I, II, and III skeletal malocclusions in an Indian adult population aged 18-25 years.

## Materials and methods

**Figure 1:**
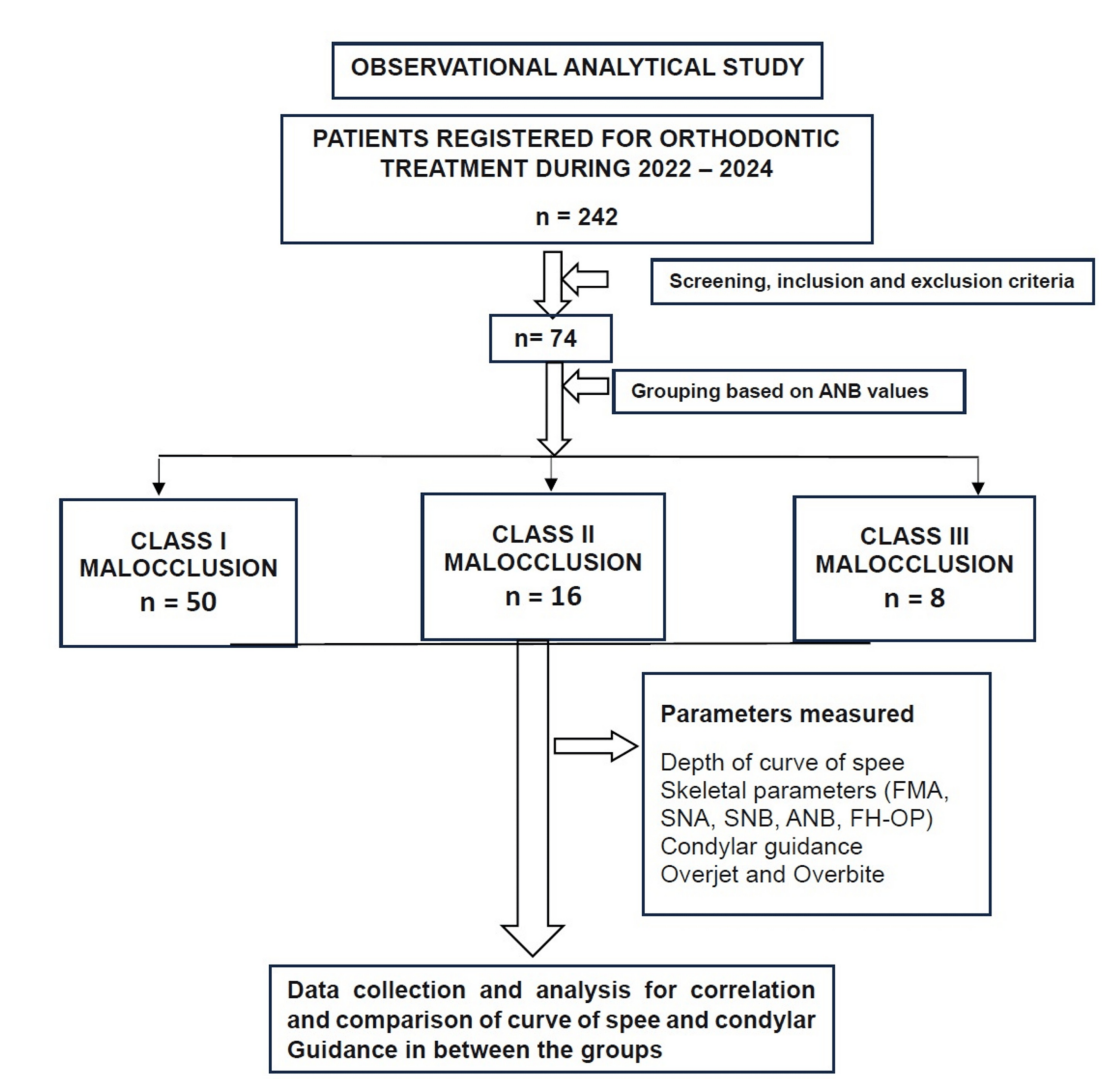
Flowchart depicting the methodology of the study

This observational study was conducted in the Department of Orthodontics, Narayana Dental College, Nellore, India, on patient records from June 2022 to December 2024. Ethical clearance was obtained from the Institutional Ethics Committee, Narayana Dental College and Hospital (ICE/NDCH/2024/MAY-AUG/P-77). The sample size was determined using G*Power version 3.1.9.6 (Heinrich Heine University Düsseldorf, Germany), with an effect size (f) of 0.5 representing a medium effect size, α = 0.05, and power = 0.80. The group allocation was based on the epidemiological distribution of malocclusion types in the Indian population, as reported by Kaur et al. [17], with Class I accounting for 66-70%, Class II for 22-25%, and Class III for 5-10%. Based on this, a minimum total sample size of 74 participants was calculated and distributed in a 6:2:1 ratio across Class I, II, and III malocclusion groups. Skeletal classification was done using ANB angle criteria: Class I (ANB 0-4°), Class II (ANB >4°), and Class III (ANB <0°). Data for this study were collected from retrospective records of patients who underwent orthodontic treatment at the department in the given period (Figure 1).

Eligible participants were aged 18-25 years and had symmetrical facial profiles, vertical overlap of incisors, and full permanent dentition (excluding third molars). Mild anterior crowding and spacing, each less than 3 mm, were accepted as per Little’s Irregularity Index [18]. Subjects were excluded if they exhibited facial asymmetry, craniofacial syndromes, open bite, crossbites, retroclined upper incisors, periodontal disease, dental anomalies, prosthetic restorations, TMJ disorders, trauma, or had undergone prior orthognathic surgery. Out of 242 available pretreatment records, 74 subjects were selected after applying specific inclusion and exclusion criteria and were assigned to three groups according to skeletal and molar relationships corresponding to Class I, Class II, and Class III malocclusion patterns.

The methodology for this study was derived and slightly modified from previously established protocols [5,15], with adjustments tailored to the study’s specific objectives and sample characteristics. Records included study models, lateral cephalograms, and orthopantomograms (OPGs). Digital images were obtained using standardized machines (Villa Sistemi, Planmeca Promax). Models were scanned as STL files (SHINING 3D AutoScan-DS-EX; SHINING 3D, Hangzhou, China). Images were calibrated using the first molar width for 1:1 magnification. All linear and angular measurements were carried out using Dolphin Imaging software (Premium version 11.95, Dolphin Imaging & Management Solutions, Chatsworth, CA, USA).

**Figure 2:**
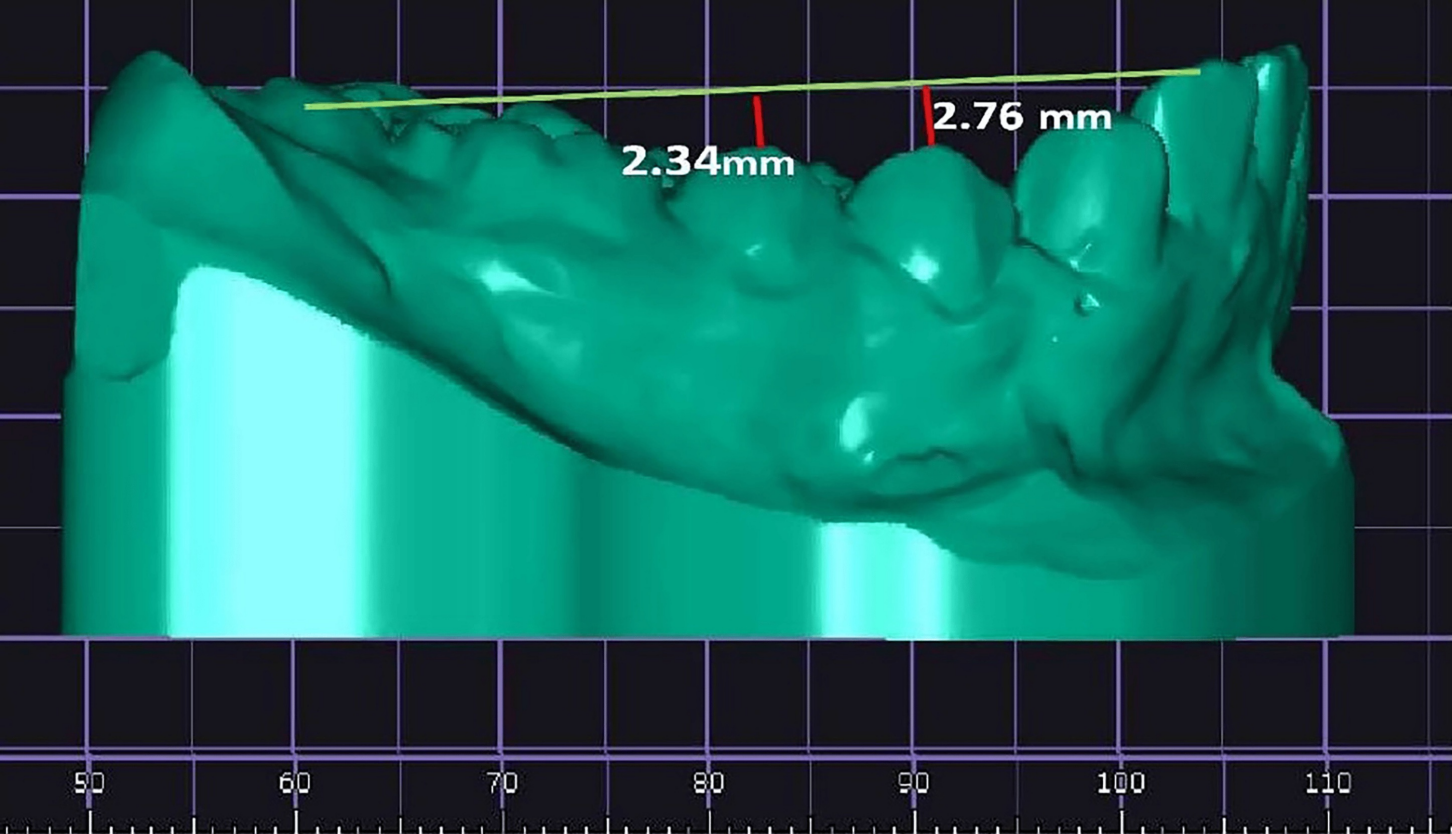
Measurement of the curve of Spee (COS) on digital models

The primary parameter, COS was measured on scanned mandibular models as the perpendicular distance from the deepest cusp tip of either premolar to the constructed occlusal plane drawn from the incisal edges of the mandibular central incisors to the distal cusp tips of the last erupted molars (functional occlusal plane or best-fit plane) (Figure 2).

**Figure 3:**
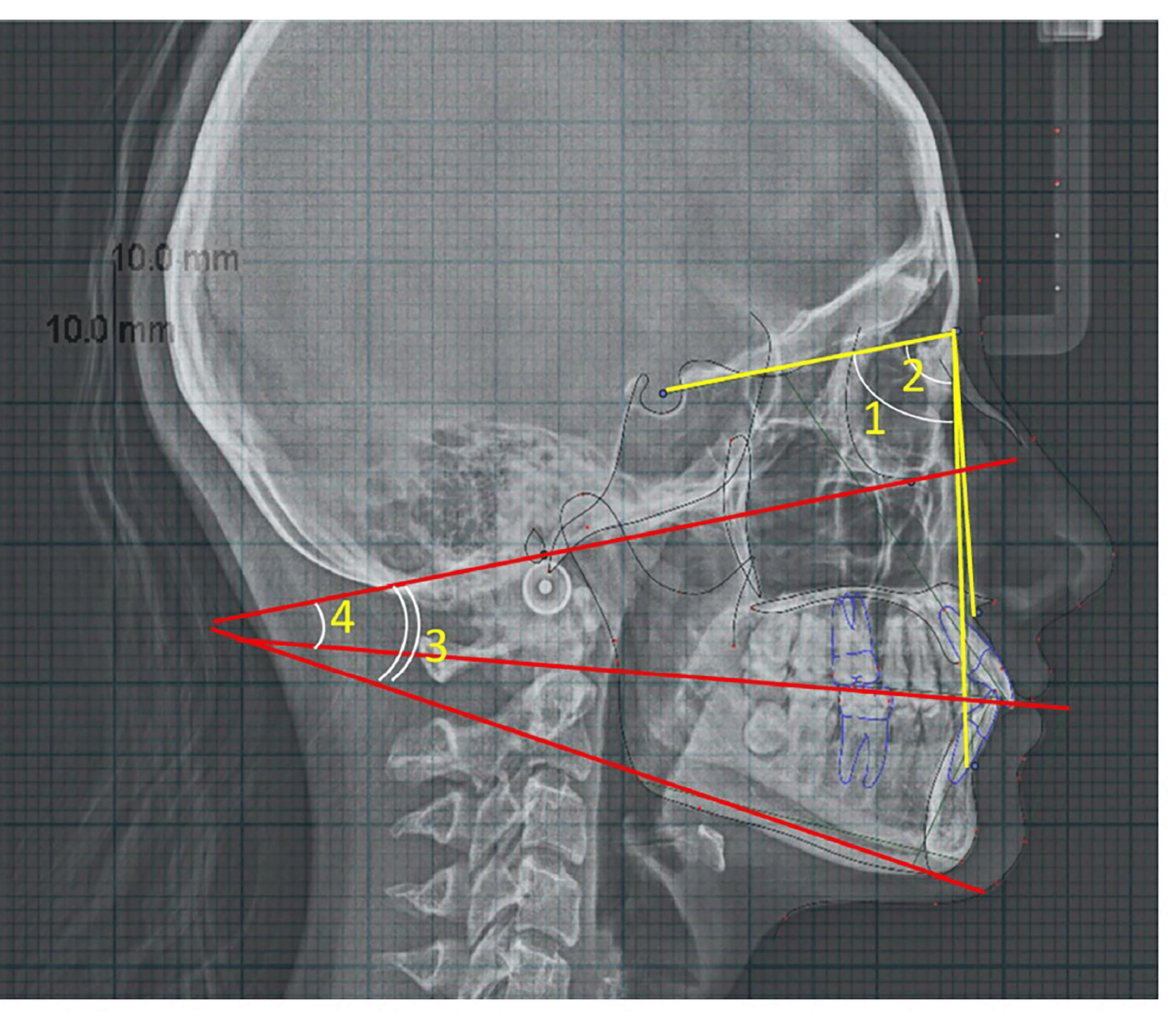
Skeletal parameters measured on lateral cephalogram 1. SNA: Sella-Nasion-point A (angle); 2. SNB: Sella-Nasion-point B (angle); 3. FMA: Angle between Frankfurt horizontal-mandibular plane; 4. FH-OP: Angle between Frankfurt horizontal and occlusal plane

**Figure 4:**
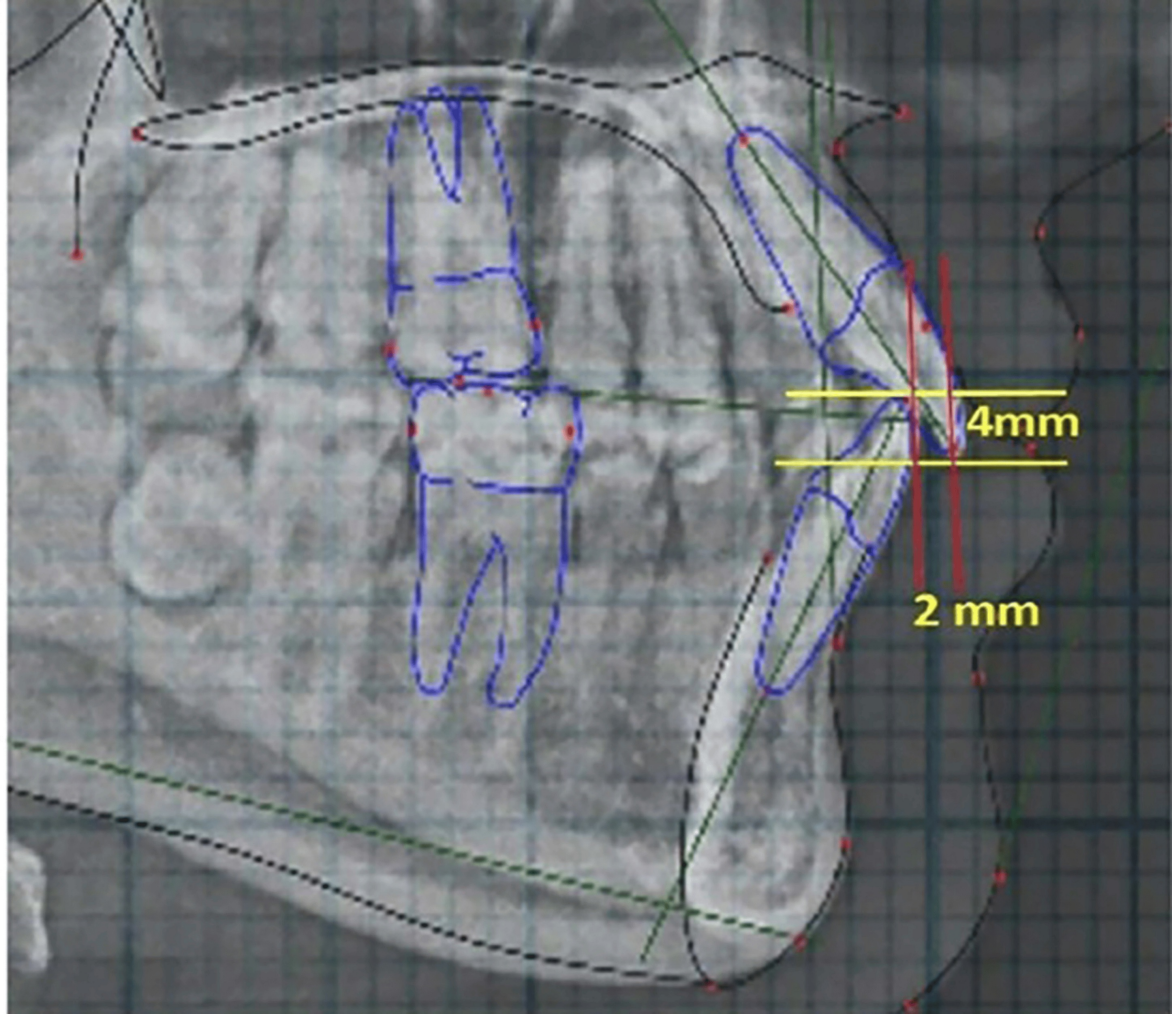
Measurement of overjet and overbite on lateral cephalogram

**Figure 5:**
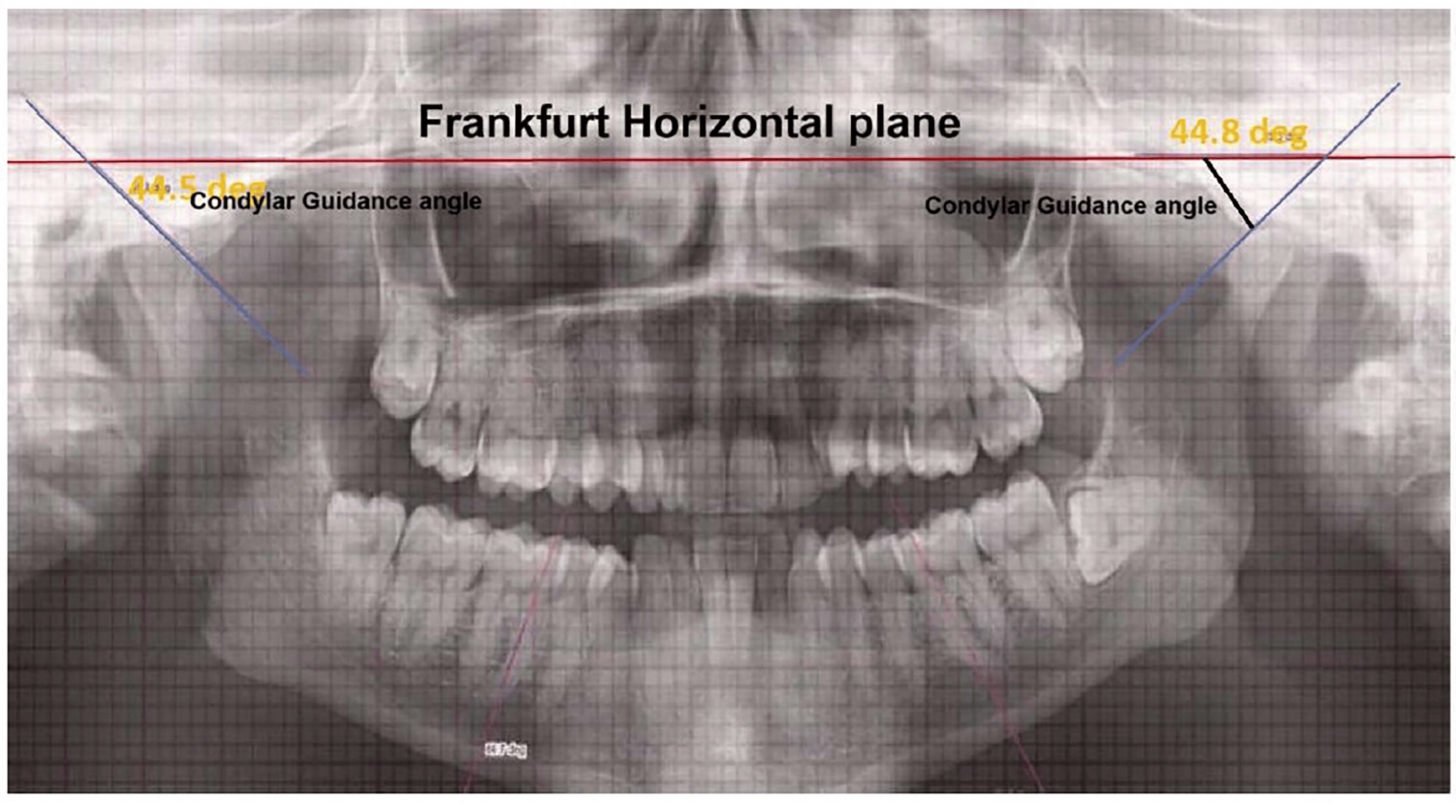
Condylar guidance (CG) – the angle between the Frankfurt Horizontal (FH) plane and constructed condylar path on orthopantomogram (OPG)

Cephalometric parameters included SNA: Sella-Nasion-point A (angle); SNB: Sella-Nasion-point B (angle) to assess sagittal skeletal relationships, along with the Frankfurt Mandibular Plane Angle (FMA) and the angle between the Frankfurt Horizontal (FH) plane and the occlusal plane (FH-OP) (Figure 3). Dental measurements, such as overjet and overbite, were obtained from digitized lateral cephalograms using standardized landmarks (Figure 4). CG was assessed on the OPG by measuring the angle between the FH plane and the constructed condylar path, following the protocol suggested by Mawani et al. (Figure 5) [5].

The primary investigator (MMT) underwent a one-month calibration under expert supervision (SGS), achieving ≥98% ICC consistency using Dolphin Imaging. Methodological reliability was confirmed through repeated tracings of 20 cephalograms after a two-week interval, with intra- and inter-method ICC values exceeding 0.90 for both linear and angular measurements, indicating excellent reproducibility. The values of the primary researcher were taken for the analysis.

Statistical analysis

The collected data were compiled in Microsoft Excel (Microsoft Corporation, Redmond, WA, US) and analyzed using the DATAtab Online Statistics Calculator (DATAtab e.U., Graz, Austria). Continuous variables were expressed as means and standard deviations. Data normality was evaluated using the Kolmogorov-Smirnov test with Lilliefors correction. Pearson’s correlation coefficient was utilized to evaluate the relationship between the dependent variable, COS, and the independent continuous variables. Differences among malocclusion groups were examined using one-way analysis of variance (ANOVA) and the student’s t-test. A p-value of <0.05 was considered statistically significant.

## Results

There were no statistically significant differences in age or gender distribution among the groups (Table 1). Descriptive statistics revealed that the mean COS was highest in Class II (2.98 ± 0.50 mm), followed by Class I (2.41 ± 0.61 mm), and lowest in Class III (1.75 ± 0.40 mm) (Table 2). ANOVA confirmed that these intergroup differences in COS were statistically significant (p < 0.001). Similarly, the Occlusal Plane angle (FH-OP) also showed a significant variation among groups (p = 0.02), with Class III subjects presenting a notably flatter occlusal plane (Table 3). In contrast, CG showed no significant difference between the malocclusion types (p = 0.464). Post-hoc Tukey’s test revealed significant pairwise differences in COS between all groups and in FH-OP between Class III and Classes I and II (Table 4). Correlation analysis indicated a weak, statistically significant negative correlation between COS and FH-OP in Class I (r = -0.31, p = 0.03). In contrast, no significant correlation was found between COS and CG in any malocclusion group (Table 5).

**Table 1:** Basic demographic details of all three malocclusion categories * ANOVA for age and chi-square test for gender; S.D.: standard deviation; NA: not applicable; F: F-ratio; X²: chi-square statistic value; NS:’ p’ values greater than 0.05 are statistically non-significant; ANOVA: analysis of variance

Variable	Malocclusion class category	N (%)	Mean ± S.D. (in years)	Frequency	Test static*	p-value
Age	I	50 (68%)	21.34 ± 4.0	NA	F=1.26	0.29^NS^
	II	16 (23%)	20 ± 2.60			
	III	8 (9%)	19.75± 2.63			
Gender	I	50 (68%)	Male	16	X^2^=1.61	0.44^NS^
			Female	34		
	II	16 (23%)	Male	6		
			Female	10		
	III	8 (9%)	Male	1		
			Female	7		

**Table 2:** Descriptive parameters of different classes of malocclusion S.D.: Standard Deviation; C.I.: Confidence Interval; SNA^(o)^: Sella-Nasion-Point A in degrees; SNB^(o)^: Sella-Nasion-Point B in degrees; ANB^(o)^: Difference between SNA and SNB in degrees; OJ: Overjet in mm; OB: Overbite in mm; COS: Curve of Spee in mm; FMA^(o)^: Frankfurt Horizontal: Mandibular Plane Angle in degrees; FH-OP^(o)^: Frankfurt Horizontal-Occlusal Plane Mandibular Plane Angle in degrees; ANOVA: analysis of variance

Parameter	Class I (n=50)		Class II (n=16)		Class III (n=8)	
	Mean ±S. D	95% CI	Mean ±S. D	95% CI	Mean ±S. D	95% CI
SNA(^o^)	82.4±2.54	81.68 - 83.12	82.88±3.2	81.17 - 84.58	81.38±2.77	79.06 - 83.69
SNB (^o^)	79.68±2.63	78.93 - 80.43	77.31±3.26	75.58 - 79.05	83.5±3.63	80.47 - 86.53
ANB (^o^)	2.72±1.26	2.36 - 3.08	5.56±1.31	4.86 - 6.26	-2.12±2.1	-3.51-1.13
OJ (mm)	2.55±1.26	2.2 - 2.91	4.05±0.55	3.76 - 4.34	1.38±0.52	0.94 - 1.81
OB (mm)	2.59±1.32	2.22 - 2.96	2.71±1.11	2.11 - 3.3	1.38±0.52	0.94 - 1.81
CG (^o^)	45.25±6.73	43.34 - 47.17	43.43±7.61	39.37 - 47.48	46.9±5.24	42.52 - 51.28
COS (mm)	2.41±0.61	2.24 - 2.58	2.98±0.5	2.72 - 3.25	1.75±0.4	1.41 - 2.09
FMA (^o^)	22.76±4.02	21.62 - 23.9	24.69±5.34	21.84 - 27.53	23.88±4.39	20.2 - 27.55
FH-OP (^o^)	13.09±3.44	12.11 - 14.06	12.55±1.77	11.61 - 13.49	9.82±1.5	8.57 - 11.08

**Table 3:** Comparison of condylar guidance (CG), curve of Spee (COS), and the occlusal plane (FH-OP) between the different malocclusions: analysis of variance (ANOVA) I: class I malocclusion; II: class II malocclusion; III: class III malocclusion; S.D.: standard deviation; MS: mean square; SS: sum of square; df: degrees of freedom; * ‘p’ values less than 0.05 are statistically significant

Parameter/Variable	Class of malocclusion		Mean ± SD	ANOVA summary						F-ratio	p-value
				Factor			Residual				
				df	MS	SS	df	MS	SS		
Condylar Guidance (CG)	CG-I	n=50	45.25 ± 6.73	2	35.85	71.7	71	46.21	3280.99	0.78	0.464
	CG-II	n=16	43.43 ± 7.61								
	CG-III	n=8	46.9 ± 5.24								
Occlusal Plane (FH-OP)	FH-OP -I	n=50	13.09 ± 3.44	2	36.72	73.43	71	9.03	641.3	4.06	0.02*
	FH-OP -II	n=16	12.55 ± 1.77								
	FH-OP -III	n=8	9.82 ± 1.5								
Curve of Spee (COS)	COS -I	n=50	2.42 ± 0.61	2	4.28	8.56	71	0.33	23.15	13.12	< 0.001
	COS -II	n=16	2.98 ± 0.95								
	COS -III	n=8	1.75 ± 0.4								

**Table 4:** Inter-pair comparison of condylar guidance (CG), curve of Spee (COS), and the occlusal plane (FH-OP) between the different malocclusions: post-hoc Tukey test I: class I malocclusion; II: class II malocclusion; III: class III malocclusion; * ‘p’ values less than 0.05 are statistically significant.

Parameter/Variable	Interpair comparison		Mean difference (A-B)	p-value
	(A)	(B)		
Condylar Guidance (CG)	CG-I	CG-II	1.83	0.375
	CG-I	CG-III	1.65	0.451
	CG-II	CG-III	3.48	0.323
Occlusal Plane (FH-OP)	FH-OP-I	FH-OP-I	0.54	0.647
	FH-OP-I	FH-OP-III	3.26	< 0.001
	FH-OP-II	FH-OP-III	2.73	0.033*
Curve of Spee (COS)	COS-I	COS-II	0.57	< 0.001
	COS-I	COS-III	0.66	< 0.001
	COS-II	COS-III	1.23	< 0.001

**Table 5:** Correlation showing the association between the curve of Spee and other variables in all three classes of malocclusion r: Pearson correlation coefficient; I: class I malocclusion; II: class II malocclusion; III: class III malocclusion; * ‘p’ values less than 0.05 are statistically significant SNA^(o)^: Sella-Nasion-Point A in degrees; SNB^(o)^: Sella-Nasion-Point B in degrees; ANB^(o)^: Difference between SNA and SNB in degrees; OJ: Overjet in mm; OB: Overbite in mm; COS: curve of Spee in mm; FMA^(o)^: Frankfurt horizontal: mandibular plane angle in degrees; FH-OP^(o)^: Frankfurt Horizontal-Occlusal plane mandibular plane angle in degrees

Parameter	COS-I		COS-II		COS III	
	r	p	r	p	r	p
SNA	0.1	0.493	0.05	0.851	-0.1	0.82
SNB	0.02	0.892	-0.05	0.859	0.07	0.862
ANB	0.16	0.272	0.24	0.361	-0.25	0.543
OJ	-0.21	0.148	-0.42	0.106	-0.17	0.684
OB	-0.15	0.293	0.41	0.114	-0.34	0.404
CG	-0.1	0.486	0.28	0.289	-0.34	0.415
FMA	0.04	0.756	-0.32	0.222	-0.16	0.701
FH-OP	-0.31	0.031	0.05	0.842	-0.41	0.313

## Discussion

The COS is a critical occlusal parameter influencing functional efficiency, overbite correction, and long-term orthodontic stability. Its role in treatment planning is particularly significant due to its variability across malocclusion types and its impact on anchorage and occlusal leveling. CG, a fixed anatomical determinant of mandibular movement, influences mandibular dynamics and is thought to affect occlusal morphology indirectly [4,5].

CG, a fixed anatomical determinant guiding mandibular movement, is believed to influence occlusal relationships indirectly. However, the extent to which CG contributes to COS development remains unclear. While several studies have evaluated COS in relation to overjet, overbite, and ANB angles, only one study has directly assessed its correlation with CG, and none of the studies in the literature have evaluated this relation across malocclusion types [15]. Understanding this relationship is important in orthodontics, as it may inform treatment planning decisions, particularly in cases requiring COS leveling for bite correction in different skeletal malocclusions.

In the present study, Class II subjects showed the highest COS values (2.98 mm), followed by Class I (2.42 mm) and Class III (1.75 mm) (Table 2). These results align with multiple previous studies that consistently report deeper COS in Class II malocclusion. For instance, Shannon & Nanda and Baydas observed significantly greater COS in Class II than in Class I or III [19,20]. A study by Nayar et al. similarly reported mean COS values above 2.7 mm for Class II patients, with shallower values in Class I (around 2.2-2.3 mm) in the Indian population [21], and another study by Aboulfotouh and El-Dawlatly has echoed similar trends in the Egyptian population [22]. In the current study, the Class III group consistently displayed the flattest COS, a finding also supported by earlier studies [7,9], suggesting that anterior mandibular positioning restricts the deepening of the curve.

Understanding the interplay between the COS, occlusal plane inclination (FH-OP), and condylar guidance (CG) is critical in orthodontics, particularly when designing biomechanically efficient and stable treatment plans. While COS and FH-OP are dynamic and routinely altered during orthodontic mechanotherapy, CG is an anatomic constant. This study also evaluated whether these parameters vary across different sagittal skeletal malocclusions (Class I, II, III) and if any meaningful associations exist that could influence clinical decision-making.

The ANOVA results (Table 3) showed a statistically significant difference in COS (p < 0.001) and FH-OP (p = 0.02) across the malocclusion groups. The post-hoc Tukey test (Table 4) revealed that Class II subjects had a significantly deeper COS than Class I and III, and Class III had the flattest occlusal plane (lowest FH-OP angle). These findings suggest a compensatory dentoalveolar adaptation in Class II (exaggerated COS and steep OP) and a flatter occlusal scheme in Class III, likely due to skeletal mandibular protrusion.

In contrast, CG did not differ significantly between groups (p = 0.464), and post-hoc comparisons showed no statistically significant pairwise differences. This confirms that CG is independent of sagittal skeletal classification and does not directly influence COS, supporting previous literature (Table 4).

Despite clear group differences in COS, correlation analyses revealed no statistically significant associations between COS and skeletal angles (SNA, SNB, ANB) across the malocclusion classes (Table 5). Cheon et al. reported significant positive correlations between COS and SNB (r = 0.38) and ANB (r = 0.427) [8], whereas Patil et al. reported weak, non-significant correlations [7], similar to our findings. In our Class III cohort, ANB showed a weak negative correlation with COS (r = -0.25), indicating a flatter curve with more pronounced mandibular prognathism, in agreement with Batham and Bernstein [6,23]. The lack of association between CG and COS reinforces the idea that COS is more of an adaptive response than a direct function of condylar mechanics. This aligns with the research of Owen, which emphasized that incisal guidance, not CG, is modified during deep bite correction [4].

As an anatomic constant, condylar guidance is thought to influence mandibular path movement but is not directly modifiable during orthodontic treatment. In the current study, the mean CG was highest in Class III (46.9°), followed by Class I (45.25°) and Class II (43.43°), but these differences were not statistically significant (Table 1, Table 3). No strong or significant correlations were found between CG and COS, regardless of malocclusion type. Mawani et al. noted that CG values typically range between 22-60° [5], which aligns with the values observed here. Harini et al. reported similar CG in Class II Div 1 subjects (43.74° ± 6.25), matching the present Class II findings [15].

A significant negative correlation between the FH-OP angle and COS was observed in Class I (r = -0.31, p = 0.03), suggesting that a flatter occlusal plane is associated with deeper COS (Table 5). This is functionally relevant, as COS leveling during orthodontic treatment often leads to occlusal plane alteration. This relationship has been supported by a previous study by Fawaz et al., who noted changes in occlusal plane inclination associated with COS flattening [16].

While several studies, including Batham et al., Veli et al., Baydas et al., and Kumari et al. have documented significant positive correlations between COS and overjet/overbite, our study did not replicate these findings [6,9,20,24]. In the present dataset, overjet had a weak negative correlation with COS across all classes, while overbite had a weak positive correlation only in Class II, neither reaching statistical significance.

The rationale for comparing these parameters across malocclusions lies in their functional interdependence during occlusion. A deeper COS often necessitates more aggressive leveling mechanics, which could potentially disrupt the harmonious interaction between anterior guidance and posterior disclusion, especially if CG is steep. Conversely, flatter occlusal schemes, as seen in Class III, may demand different biomechanical strategies.

By identifying that COS and FH-OP vary significantly by skeletal class, but CG does not, the study reinforces the idea that COS and the occlusal plane are adaptive, treatment-modifiable features, while CG remains a constant skeletal guide. This differentiation is critical when planning leveling and bite-opening mechanics, particularly in cases requiring functional occlusal rehabilitation.

A notable strength of this study is its inclusion of all three skeletal malocclusion types with standardized imaging protocols and measurement calibration. It is among the first to compare COS and CG in natural dentitions. Notwithstanding its merits, this study possesses specific limitations: the Class III malocclusion cohort had a low sample size (n = 8), potentially constraining statistical power and the capacity to identify nuanced variations in condylar guidance or occlusal traits. Condylar guiding was evaluated using OPGs, which, as a two-dimensional imaging technique, may lack the accuracy of three-dimensional modalities like CBCT. Vertical skeletal discrepancies (hypo-, normo-, and hyperdivergent patterns) were not analyzed individually, despite their known impact on the COS and occlusal plane orientation. The retrospective design and single-institution context may restrict the applicability of findings to wider groups.

Future research should utilize larger, balanced samples, particularly for Class III malocclusions, to improve statistical validity. Integrating 3D imaging methods would provide a more precise evaluation of condylar guiding and craniofacial interactions. Longitudinal studies are required to assess alterations in COS and CG during orthodontic treatment and post-treatment retention.

## Conclusions

This study found that while the curve of Spee (COS) varies significantly across different sagittal skeletal malocclusions, being deepest in Class II and shallowest in Class III, no significant correlation exists between condylar guidance and COS. These findings suggest that the COS may be influenced more by adaptive dentoalveolar responses and occlusal plane inclination rather than fixed skeletal parameters like condylar guidance. Future research with larger, stratified samples and inclusion of vertical and transverse skeletal patterns is warranted to delineate these relationships better.
